# Costs of outpatient and inpatient MRSA screening and treatment strategies for patients at elective hospital admission - a decision tree analysis

**DOI:** 10.1186/s13756-018-0442-x

**Published:** 2018-11-29

**Authors:** Luise Hutzschenreuter, Steffen Flessa, Kathleen Dittmann, Nils-Olaf Hübner

**Affiliations:** 1 Institute of Health Care Management, University of Greifswald, Friedrich-Loeffler-Str. 70, 17489 Greifswald, Germany; 2 Institute of Hygiene and Environmental Health, University Medicine of Greifswald, Walther-Rathenau-Straße 49a, 17489 Greifswald, Germany; 3 IMD Laboratory Greifswald MVZ GmbH, Vitus-Bering-Straße 27a, 17493 Greifswald, Germany

**Keywords:** Methicillin-resistant *Staphylococcus aureus*, Outpatient screening, Decolonization, Admission screening, Costs, Expected costs, Decision tree analysis

## Abstract

**Background:**

Nosocomial infections are among the most common complications in hospitals. A major part is caused by multidrug-resistant organisms (MDRO). MRSA is still the most prominent and frequent MDRO. The early detection of carriers of multidrug-resistant bacteria is an effective measure to reduce nosocomial infections caused by MDRO. For patients who are planning to go to the hospital, an outpatient screening for MDRO and pre-hospital decolonization is recommended. However, the effectiveness of such pre-admission MDRO management in preparation for a planned hospital stay has not yet been sufficiently scientifically examined from an economic perspective.

**Methods:**

A decision tree will be used to develop scenarios for MDRO screening and treatment in the context of the outpatient and inpatient sectors using MRSA-positive patients as an example. Subsequently, the expected costs for the respective strategy are presented.

**Results:**

The decision tree analysis shows that the expected costs of outpatient MRSA management are €8.24 and that of inpatient MRSA management are €672.51.

**Conclusion:**

The forward displacement of the MRSA screening to the ambulatory sector and any subsequent outpatient decolonization for patients with a planned hospitalization is the most cost-effective strategy and should become a standard benefit. Excluding opportunity costs, the expected costs of inpatient MRSA management are €54.94.

## Background

Nosocomial infections are among the most common complications in German hospitals, and are caused by an increasing proportion of multidrug-resistant organisms (MDRO) [1]. A key measure for the control of MDRO is the early detection of carriers (screening) to initiate appropriate infection control measures, suppression therapy and adequate antibiotic therapy. MDRO screening has the potential to increase patient safety and reduce the transmission risk of the pathogen to fellow patients, thus reducing the cost of hospitalized MRSA treatment. So far, sectoral boundaries between health care providers have been a major barrier to efficient solutions. Screening for MDRO carriers in preparation for a planned hospital stay (e.g. for elective surgery) is not performed in Germany, since the necessary structures are missing and the effects are not sufficiently scientifically proven. Studies in ambulatory surgery shows benefits of preventative MRSA measures [2].

Using the multidrug-resistant organism MRSA (Methicillin-Resistant *Staphylococcus aureus*) as an representative of MDRO, a large number of studies have shown that inpatient decolonization treatments of high-risk patients lead to additional financial burdens of hospitals, for example, by extending the length of stay and higher costs for hygiene management. Studies describes MRSA-attributed costs of €6000 to €10,000 per patient case. [3–5]. Due to the paucity of literature on the costs of outpatient MRSA screening and a possible subsequent outpatient decolonization in preparation for inpatient hospitalization a comparison of outpatient and inpatient screening has to date been impossible. Due to reimbursement problems pre-admission MRSA-screening is usually not performed and a good calculation of costing data is required to perform an economic screening. The present study is intended to close this gap. From a health-economics perspective, the aim of the study is to calculate the expected costs of pre-admission MRSA treatment and inpatient MRSA management.

The study is part of the PRIME project initiated by the MDRO-network KOMPASS e. V., which introduced pre-admission MRDO screening using the example of MRSA as a model in the north-eastern part of Germany (Mecklenburg-Vorpommern). The project is funded by the Ministry of Economics, Construction and Tourism Mecklenburg-Vorpommern.

## Methods

The purpose of this study was to calculate the expected costs of the outpatient and inpatient MRSA treatment strategy for elective hospital admission using a decision tree analysis. Therefor a mixed methods were used for collecting all the necessary data. The first step of developing the decision tree was to collect data during inpatient and subsequently outpatient MRSA screenings in an own survey. The data of outpatient and inpatient MRSA decolonization treatments used were obtained from previously published studies. A decision tree developed thereafter was backed by these values.

### Definition of screening and successful decolonization

In the context of this research study, MRSA screening means a targeted anamnesis of risk factors (for all patients) and, if one or more risk factors were present, a microbiological analysis by means of a swab test. It is therefore a two-stage screening process.

The definition of risk factors are guided by the recommendations of the german Commission for Hospital Hygiene and Infection Prevention (KRINKO). Following risk factors were defined for MRSA colonization:

- *patients with known MDRO history*
- *hospitalization abroad*
- *moving from elderly-care facility or chronically care dependency*
- *patients with contact to MDRO carrier during a preceding hospital stay (*e.g. *as a room-mate)*
- *patients with hospitalization (> 3 days) or treatment in an intensive care unit in the preceding 12 months*
- *antibiotic treatment in the preceding 12 months*
- *patients with work-related contact to animals in agricultural animal fattening*
- *presence of a catheter, tracheal cannula* etc.
- *dialysis patients*
- *patients with skin ulceration, gangrene, chronic wound, deep tissue infection*
- *patient is not able to provide information*

If patients have risk factors, swabs were taken from the nose and throat combined and possibly from existing wounds.

A successful decolonization means that after the completion of a decolonization cycle (eradication with local antiseptic treatment of nose (Mupirocin) and throat, whole antiseptic body wash, etc.) and two days break, swabs are taken of all defined predilection sites at three consecutive days and all findings are negative. The decolonization is carried out according to a standardized procedure. The procedure is the same in both outpatient and inpatient settings.

### Collection of real data

In a general hospital, personnel and material costs for MRSA screening were collected over a two-week period as part of the admission screening of patients. A second MRSA screening was carried out in the outpatient sector. Based on this, the cost calculation for the screening took place.

### Personnel costs

In the first step all MRSA-attributed screening processes were identified. Afterwards the costs per minute were calculated by dividing annual personnel costs (average gross wage in accordance with the collective agreement plus employer contributions) by annual working time. This resulted in carer staff costs of €0.41 per minute.

### Material costs

The cost of personal protective equipment (disposable gloves) was based on the average consumption amount, which was valued at the hospital’s purchase price. The costs of the swabs were not included in the analysis. These were provided by the external laboratory and are part of the laboratory’s service.

### Laboratory costs

For this cost analysis, the calculation of laboratory costs was based on the scale of charges (GOP) of the German uniform valuation standard for outpatient physician services (EBM), as the laboratory services were provided by an external laboratory. GOP 30954 (targeted MRSA detection on chromogenic selective medium) was used with a value of €5.32 per test. It was assumed that the hospital and the doctor’s office incurred costs of this amount for laboratory services per screening.

### Decision tree analysis

The basis of the analysis is a multi-level decision tree with the expected costs of alternative MRSA screening and decolonization strategies in patients who are faced with a planned hospitalization. The first and most authoritative decision is whether the MRSA screening takes place on an outpatient basis, meaning at the referring physician’s office, or upon hospital admission of the patient. In this study, a two-stage MRSA screening (step 1: screening for risk factors; step 2: swab in high-risk patients) is assumed. To detect MRSA, conventional cultural cultivation of the test material was chosen.

The decision tree analysis is based on the following assumptions:

- The probability of exhibiting MRSA risk factors or being MRSA carriers is the same for patients admitted to hospital admission and before being admitted to the outpatient area.
- Patients who have been screened at hospital admission are preemptively isolated for 48 h until the findings are available.
- The unit has a high occupancy rate and two-bed rooms for the calculation of the costs per locked bed (opportunity costs).
- In the absence of risk factors for MRSA or a negative result, the path of the decision tree ends and there are no additional costs.
- In this model we supposed that one decolonization cycle is necessary for successful MRSA eradication.

The decision tree contains rectangles for decisions, circles for possibilities (these are to be placed with probabilities) and triangles for the end of a branch (path). To determine the optimal strategy in the decision tree, the rollback method was chosen. Accordingly, the optimal strategy comprises that sequence of alternative courses of action which leads to the minimum expected value. The strategy with the lowest expected costs will be sought.

The expected costs were calculated in 2 steps. All of the parameters collected and used are defined in Table 1.

**Table 1:** Description and quantification of parameters

Parameter	Description	Value	References
*p*_*R*+_	Probability of having risk factors for MRSA	72.5%	own elicitation
*p*_*MRSA*+_	MRSA prevalence in high-risk patients	3.94%	[8]
*C*_*ris*_	Costs of screening for risk factors	€0.48	own elicitation
*C*_*sc*_	Costs for swabs, documentation and laboratory testing as part of the screening (swab test)	€7.09	own elicitation
*C*_*pre* _ *iso*_	Opportunity costs for a locked bed during preemptive isolation per day	€328.36	own elicitation
*T*_*pre* _ *iso*_	Time in which the patient is preemptively isolated (in days)	2 days	see assumption
*C*_*dec* _ *hos*_	Costs for decolonization (hygienic management (workload + materials) and laboratory) in the hospital per case	€1726.66	[4]
*C*_*iso* _ *hos*_	Opportunity costs for a locked bed during decolonization per day	€328.36	own elicitation
*T*_*iso* _ *hos*_	Time in which the patient is isolated during decolonization (in days)	15.08 days	[8]
*C*_*dec* _ *out*_	Costs of outpatient decolonization per case	€91.77	[10]

First, the respective costs per path were calculated for the scenarios of MRSA management (paths A to F) presented in the decision tree. The respective formulae are shown in Table 2.

**Table 2:** Formulas to calculate the total cost per path in the decision tree

Path	Description	Formula
A	Patient without risk factors (outpatient)	*C*_*A*_ = *C*_*ris*_
B	Risk patient with negative MRSA findings (outpatient)	*C*_*B*_ = *C*_*ris*_ + *C*_*sc*_
C	Patients screened and decolonized on an outpatient basis, followed by inpatient admission	*C*_*C*_ = *C*_*ris*_ + *C*_*sc*_ + *C*_*dec* _ *out*_
D	Patient without risk factors (inpatient)	*C*_*D*_ = *C*_*ris*_
E	Patient screened in hospital, preemptive isolation to findings, result: MRSA negative	*C*_*E*_ = *C*_*ris*_ + *C*_*sc*_ + *C*_*pre* _ *iso*_ · *T*_*pre* _ *iso*_
F	Patient screened in hospital, preemptive isolation, result: MRSA positive, then isolated and decolonized in hospital	*C*_*F*_ = *C*_*ris*_ + *C*_*sc*_ + *C*_*pre* _ *iso*_ · *T*_*pre* _ *iso*_ + *C*_*dec* _ *hos*_ + *C*_*iso* _ *hos*_ · *T*_*iso* _ *hos*_

*Legend: C*_*A*_ *= Costs of path A in the decision tree, C*_*B*_ *= Costs of path B in the decision tree, C*_*C*_ *= Costs of path C in the decision tree, C*_*D*_ *= Costs of path D in the decision tree, C*_*E*_ *= Costs of path E in the decision tree,* C_F_ *= Costs of path F in the decision tree*

In a second step, the expected costs E(x) of the outpatient (out) and inpatient (hos) MRSA management alternatives were calculated using the rollback method. Based on the costs per path, the following formulae were used:

$$ {E}_{out}=\left[{C}_B\bullet \left(1-{p}_{MRSA+}\right)+{C}_C\bullet {p}_{MRSA+}\right]\bullet {p}_{R+}+{C}_A\bullet \left(1-{p}_{R+}\right) $$

$$ {E}_{hos}=\left[{C}_E\bullet \left(1-{p}_{MRSA+}\right)+{C}_F\bullet {p}_{MRSA+}\right]\bullet {p}_{R+}+{C}_D\bullet \left(1-{p}_{R+}\right) $$

### Level of analysis

The level of analysis examines the effects caused by the variation of an input parameter. In this study, the influence of the parameter rate of spatial isolation on the expected costs of the inpatient MRSA management strategy was evaluated. A given parameter was varied in three steps:

- Scenario 1: This is the baseline scenario in the decision tree, meaning that the costs of preemptive isolation and the isolation while decolonization were considered.
- Scenario 2: The preemptive isolation of screened patients until the results are available has been omitted. In case of positive MRSA findings, the patient was isolated while decolonization. This means that only a part of the isolation costs have been included in the calculation of the expected costs.
- Scenario 3: The calculation of the expected costs was done without isolation costs. There were no opportunity costs associated with the MRSA management, because the patient was not spatially isolated.

Using the rollback method, the expected costs of inpatient MRSA management were calculated by varying the isolation cost parameter.

## Results

### Screening costs

Due to the equivalent procedure of inpatient and outpatient MRSA screening, the time and material effort for this is approximately the same. The calculated costs for MRSA screening are shown in Table 3. The risk factor survey takes on average 1:10 min. This results in personnel costs of €0.48 per screening of risk factors. The second step of the screening, both in the hospital and in the doctor’s office, consists of swabbing, labeling of the swab tubes, documentation and packaging of the samples for the laboratory. These activities are part of the process “swab test”. This process requires an average of 4:05 min both in the doctor’s office and in the hospital. The personnel costs are €1.67. The swabbing itself takes an average of 0:55 min. The material costs for the use of a pair of disposable gloves are €0.10. According to the GOP, costs of €5.32 are incurred for the laboratory examination. Overall, the costs of the swab test are €7.09.

**Table 3:** Screening costs

	*C*_*ris*_	*C*_*sc*_
Staff		
duration [min]	01:10	04:05
costs [€]	0.48	1.67
Material		
gloves [piece]	0	2
costs [€]	0	0.10
Laboratory		
costs [€]	0	5.32
Total costs [€]	0.48	7.09

### Decision tree paths

**Fig. 1:**
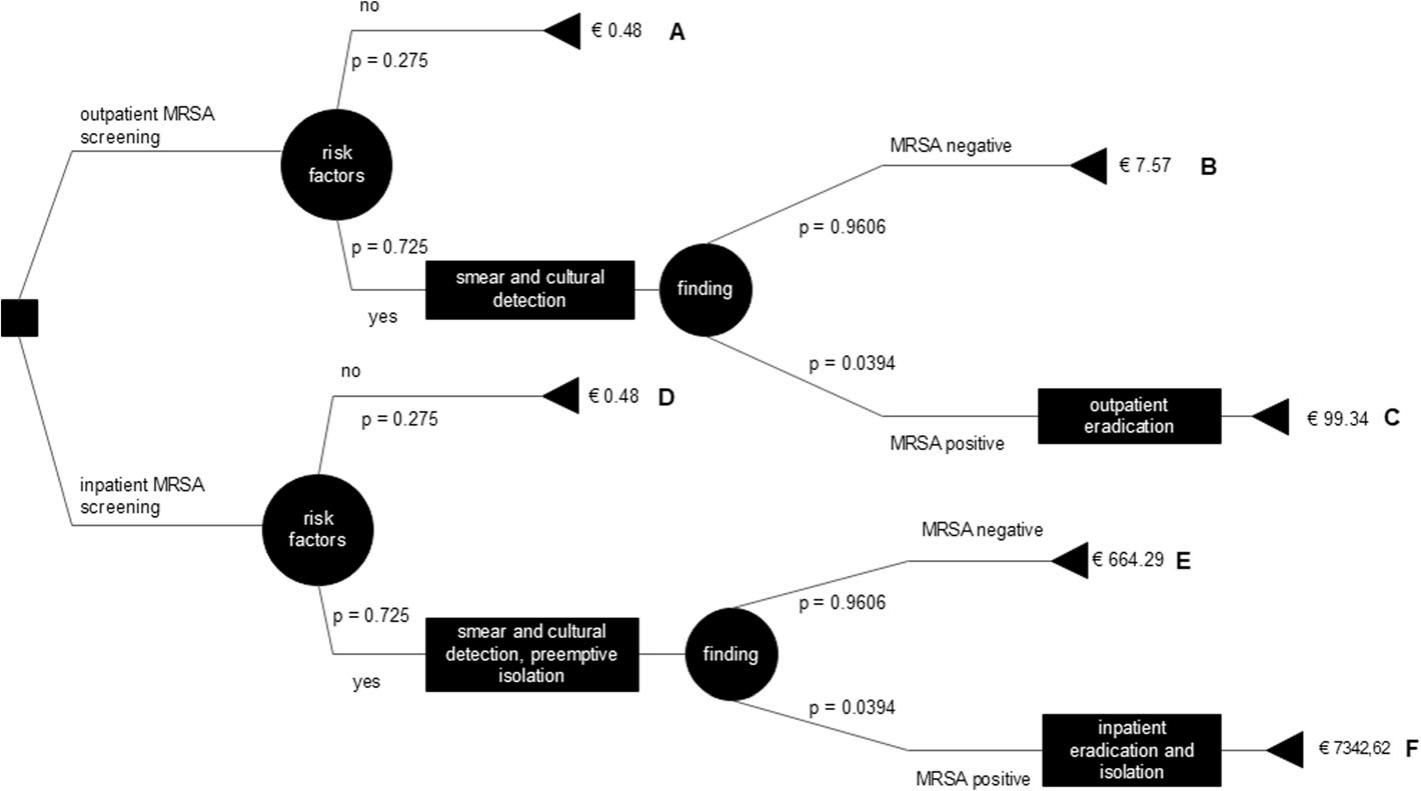
Decision tree including probabilities and total costs per path

The decision tree developed is shown in Fig. 1. Following six paths (A to F) of outpatient and inpatient MRSA management of patients with a planned hospital admission were described:

- Path A (outpatient): Patient with planned hospital admission were screened of risk factors for MRSA at an outpatient medical office. No risk factors are present, the MRSA screening is finished and hospital admission is possible.
- Path B (outpatient): Risk factors for MRSA are recorded. One or more risk factors are present. Swabs are taken from the nose and throat and if present, from wounds. Evidence is provided by culturing the material in the laboratory. After 48 h, the findings are available. The result is negative. No further measures are induced and the patient can be hospitalized.
- Path C (outpatient): Screening of MRSA and laboratory test such as path B. The findings are positive. An outpatient eradication of MRSA was implemented.
- Path D (inpatient): Patient will be screened of risk factors for MRSA at hospital admission. There are no risk factors.
- Path E (inpatient): Risk factors for MRSA are collected at hospital admission. One or more risk factors are present. Swabs are taken from defined predilection sites and microbiologically examined. Until the findings are available (after approximately 48 h), the patient is preemptively isolated on ward. The findings are negative. No further measures are induced and the patient can go to ward.
- Path F (inpatient): Screening of MRSA, laboratory test and preemptive isolation such as path E. The findings are positive. The isolation continues and in addition to initiating basic hygiene barrier measures, eradicative treatment is carried out.

In total, six possible scenarios (Paths A to F) from MRSA screening to eradication were developed (Fig. 1). In scenarios A, B and C, MRSA management takes place in the outpatient sector. Scenarios D, E and F relate to inpatient MRSA management.

### Costs calculation of paths

The decision tree also shows the costs that were calculated per path. The lowest costs are incurred with paths A and D (patient without risk factor), at €0.48. From an economic point of view, it does not matter whether the assessment of the risk factors is carried out on an outpatient or inpatient basis, as the same personnel costs arise for the collection of the risk factors in both contexts. If a follow-up examination is carried out after the identification of risk factors with a subsequent negative result, the costs in the ambulatory area (path B/€7.57) are significantly lower than those in the inpatient area (path E/€664.29). The greatest cost factor in the hospital is the two-day preemptive isolation of the patient (opportunity costs through a locked bed) until the findings are available.

If a patient is tested outpatient positive for the sponsorship of MRSA and then carried out an outpatient decolonization (path C) costs incurred by a registered doctor in the amount of €99.34. The highest costs are found in path F (patient inpatient positive for MRSA tested and then decolonized stationary) with €7342.62.

### Expected costs of the strategies

The calculation of the expected costs for outpatient and inpatient MRSA management by means of the rollback method has shown that when practicing MRSA management, the attending physician generates expected costs (E_out_) per patient of €8.24. The expected costs of the inpatient MRSA management strategy (E_hos_) are €672.51.

### Level of analysis

In the scenarios where the patient is isolated in the hospital (paths E and F), the initial situation creates opportunity costs by locking beds while isolating the patient. If the opportunity costs are ignored in whole or in part, as expected, the expected costs for the considered inpatient MRSA management strategy change. In the basic scenario (preemptive isolation and isolation given a positive finding), expected costs were €672.51 for inpatient MRSA management. If preemptive isolation of the patient is not performed until the lab results are ready (unclear MRSA status), the expected costs of the inpatient MRSA management strategy are reduced to €196.39 per patient. If a procedure is chosen without spatial isolation of the patient, the expected costs of inpatient MRSA management are €54.94 (Table 4).

**Table 4:** Expected costs of the inpatient MRSA management strategy with variable opportunity costs

Scenario	Description	Expected costs of inpatient MRSA management
1	Basic scenario (preemptive isolation and isolation if MRSA positive)	€672.51
2	No preemptive isolation of the screened patient, but isolation if MRSA positive while decolonization	€196.39
3	Inpatient MRSA management without isolation of the patient (no opportunity costs)	€54.94

## Discussion

The decision tree analysis shows that the expected costs of outpatient MRSA management (€8.24 per case) are far below those of inpatient MRSA management (€672.51 per case). Diller et al. (2008) show that pre-admission MRSA screening is cost-effective [6]. In their study, they had discovered 5 MRSA-positive patients by pre-admission screening and decolonized them before hospitalization. As a result, they could avoided costs for MRSA-treatment and isolation of approximately €30,000 to €50,000. Also Wernitz et al. (2005) demonstrated that precocious screening and if necessary decolonization of MRSA reduce costs [7]. Giese et al. (2013) calculated that a pre-admission decolonization at home in 22 cases saved a total of about €134,000 to €205,000 [8]. In conclusion, pre-admission screening and if necessary decolonization before hospital treatment is advantageous from an economic perspective.

If only MRSA screening is initially considered, a significant cost difference between outpatient (path B / €7.57) and inpatient MRSA screening (path E / €664.29) can be seen. The cost of determining the risk factors and performing the smear are identical. The enormously higher costs of inpatient MRSA screening can be explained by the costs for preemptive isolation of all patients, where were taken swabs (48 h til presence of laboratory results) in hospital. An outpatient MRSA screening does not require isolation of the patient. Therefore, there are no costs for isolation in the outpatient sector. The costs of MRSA screening are determined by the method of laboratory test. In our study a conventional cultural cultivation of the test material was chosen, which imply a preemptive isolation. If a polymerase chain reaction (PCR) method were chosen, preemptive isolation is not necessary and no additional costs for isolation are incurred [9].

The level of analysis has shown that the expected costs of inpatient MRSA management are still higher than those in the outpatient setting, if patient isolation is excluded. When considering MRSA decolonization, the different levels of costs can be attributed primarily to the extent of expenses incurred by the service provider for eradication therapy of an MRSA patient. For example, in the case of outpatient MRSA decolonization, the personnel, material and laboratory costs incurred arise when the patient has contact with the doctor’s office during eradication therapy (for example control smears, counseling session). The actual decolonization is carried out by the patient independently at home. The cost of the necessary decontamination set, consisting of antiseptic preparations such as mouthwash, washing lotion and surface disinfectant cannot be charged to statutory health insurance (SHI); this is funded by the patient him- or herself. Only the antibacterial nasal ointment can be prescribed as an authorized medicinal product covered by the SHI. The costs of an MRSA decolonization in the hospital arise from the extra work required for hygiene management (e.g., increased personnel and material costs for changing clothes), the implementation of eradication therapy, including all the preparations used to decolonize the patient, the laboratory costs and the opportunity cost of blocking beds during isolation of the patient [6]. All preparations and protective equipment necessary for treatment are provided and financed by the hospital, and no costs will be charged to the patient.

The greatest expense factor of hospital MRSA management are the opportunity costs. These arise both in the preemptive isolation of all screened patients and in the isolation of MRSA-positive patients while decolonization. However, there are also indications that in the reality of German hospitals, hardly any patients are rejected and therefore no opportunity costs are incurred [10]. This depends on the hospital’s bedload. Additional costs arise for organizing the room change when patients requiring isolation are admitted to the ward. These costs for preparing a suitable room, relocating other patients, and the related space management cannot be illustrated in this study, but should be mentioned.

### Current settlement situation of outpatient MRSA services

Until now, outpatient MRSA coverage is only provided by statutory health insurance if it concerns the further treatment of an MRSA-colonized patient after hospital discharge (on the settlement of GOP 30949 to 30952 in section 30.12 of the EBM). Expansion of the billable positions in the EBM by pre-admission screening and subsequent outpatient decolonization before hospital admission should be targeted from an economic perspective.

#### Demands for pre-admission screening

The Commission for Hospital Hygiene and Infection Prevention at the Robert Koch Institute considers an advanced MRSA screening in planned hospital admissions useful for reducing the transmission and infection risk [11]. Since 2015, the National Association of Statutory Health Insurance Physicians has also been calling for the compensation of outpatient services that are associated with pre-admission MRSA screening [12]. The aim is to curb the spread of the pathogen. Specifically, this requirement refers to a smear test in patients with risk factors who are about to undergo surgery. Several studies have proven that MRSA screening and subsequent decolonization of MRSA-positive patients prior to surgery reduces the number of postoperative wound infections and leads to cost savings [13, 14].

### Strengths and limitations

The strengths and limitations of this study should not go unmentioned. The collection of real data for MRSA screening enabled real costs to be calculated for the scenarios presented in the decision tree. For the development of the decision tree and the calculation of the expected costs of the MRSA management strategies, only a few assumptions had to be made due to the extensive data collection in MRSA screenings and the resulting data. A strength of the study is that the expected cost were calculated in dependent on the probability of occurrence. The method of roll-back analysis enables to generalize the costs of the outpatient and inpatient strategy. The use of secondary data for the cost calculation for MRSA eradication is justified in the scope of the project, which has primarily introduced a pre-admission MRSA screening and has not additionally examined the subsequent decolonization. The decision tree can only use for planned hospital admission. This decision tree cannot be applied to patients admitted as an emergency in the hospital. Another limitation is that individual characteristics of planned hospital admissions or patients cannot be taken into account in this model (eg., whether the individual patient can carry out the decolonization at home independently or needs help from a community nurse). The main strength of our study is that the developed decision tree and the cost calculation can be a decision-making aid, whether from an economic perspective a pre-admission MRSA screening or MRSA decolonization should be introduced in preparation for a planned hospitalization.

## Conclusion

The expected costs of an outpatient MRSA strategy are always lower than those of an inpatient strategy, as there is no isolation of the patient and the decolonization is performed independently by the patient at home. From the point of view of this cost analysis, pre-admission MRSA management is recommended before a planned hospital stay.

## Abbreviations

EBM: German uniform valuation standard for outpatient physician services
GOP: Scale of charges
KRINKO: The Commission for Hospital Hygiene and Infection Prevention
MDRO: Multidrug resistant organisms
MRSA: Methicillin-Resistant *Staphylococcus aureus*
SHI: Statutory health insurance
